# Implementation of a Pre-X-Ray Safety CHECK in Neonates

**DOI:** 10.1097/pq9.0000000000000860

**Published:** 2025-12-23

**Authors:** Debra Armbruster, Megan Barcroft, Michael Stenger, Chance Alvarado, Roopali Bapat

**Affiliations:** From the * Division of Neonatology, Department of Pediatrics, Nationwide Children’s Hospital NICU, The Ohio State University Wexner Medical Center, Columbus, Ohio; † Division of Neonatology, Department of Pediatrics, Nationwide Children’s Hospital, Columbus, Ohio; ‡ Biostatistics Resource at Nationwide Children’s Hospital, Abigail Wexner Research Institute, The Ohio State University Wexner Medical Center, Columbus, Ohio.

## Abstract

**Introduction:**

Radiographs are diagnostic tools used in neonatal medicine for diagnosis, reducing harm from vascular devices, and monitoring endotracheal tube placement. The lack of neonatal exposure guidelines and the repetition of radiographs due to poor quality have cumulative effects of radiation overexposure. This project aimed to improve the radiographic quality of films by 15% by using a standardized checklist before radiographic exposure, thus reducing repeat exposures.

**Methods:**

Radiographs were classified into 3 groups. The All-X-Ray Group consisted of all abdominal, baby gram, and chest films. Two subgroups included films with an indwelling device (peripherally inserted central catheter or an endotracheal tube). Data were compared from a Baseline Period through an Intervention Period and a Sustain Period (June–July 2022). Three sequential interventions were implemented: the implementation of a standardized safety check, increased unit presence, and hands-on simulation training. The primary outcome was a high-quality diagnostic film that met 4 predefined criteria. Measures were assessed using statistical process control methods through a time-series design to identify changes over time.

**Results:**

The quality of films in the All-X-Ray Group improved by 46% (19%–65%), whereas the quality of films in the peripherally inserted central catheter and endotracheal tube subgroups improved by 79% (2%–81%) and 73% (1%–74%), respectively.

**Conclusions:**

Standardization of the radiograph process led to improved quality of neonatal films in this unit. Consistent hands-on training enabled sustainable impact. Empowering bedside staff resulted in a positive culture change. A standard pre-x-ray checklist improved the diagnostic quality of films in a neonatal unit when combined with increased provider presence during the x-ray procedure and staff education.

## INTRODUCTION

Portable radiographs remain an essential diagnostic test for infants in the neonatal intensive care unit (NICU). X-rays may be required for clinical diagnosis and evaluation of vascular or respiratory devices.^1,2^ The survival of extremely preterm infants has vastly improved during the last few decades.^3^ Due to the high rate of cell turnover in premature infants, the risk of radiation dose holds significant concern.^4^ Repeating radiographs during routine care impacts the cumulative effect of the radiation dose received during a NICU stay.^5^ The potential long-term harm due to the effects of radiation dose is real; however, it is not immediately evident.^6–8^ The radiation dose risk for preterm infants exceeds the equivalent radiation dose for adults.^5–8^

Poor-quality radiographs can lead to repeat films for clarification, resulting in additional radiation exposure.^8^ Variables triggering repeat radiographs include incorrect positioning, poor exposure, head or body rotation, and wires or other artifacts that may interfere with interpretation.^6,9^ Appropriate patient positioning is essential to identify the indwelling device position.^2^ Neck position can significantly influence the endotracheal tube (ETT) position on a supine chest radiograph.^1,2^ In addition, arm and leg positioning can impact the location of the peripherally inserted central catheter (PICC).^2^ Correct positioning of the x-ray beam is also important to include the intended areas while limiting unnecessary radiation exposure to organs through the use of proper collimation or target area.^7,8,10^ Collimation impacts the radiation dose delivered with each radiograph.^7,9,11,12^ The lack of radiology practice guidelines that consider the unique anatomy of neonates has contributed to persistent radiation overexposure and inaccuracies.^4,5,9^ The specific aim of this quality improvement (QI) project was to increase the frequency of high-quality diagnostic radiographs by 15% during a 9-month period for all neonatal radiographs in our level III NICU from May 2021 to March 2022. Our overall goal was to enhance radiograph quality, thereby reducing the need for repeat imaging and minimizing the risk of additional radiation exposure.

## METHODS

### Context

The project was approved by the Neonatal QI Steering Committee of Nationwide Children’s Hospital. The setting was the 42-bed NICU, managed by Nationwide Children’s Hospital and located in Columbus, Ohio, within the The Ohio State University Wexner Medical Center (OSUWMC). The NICU is situated within the university’s adult academic facility, with a delivery service averaging 5,400 live births annually.

### Interventions

We established a multidisciplinary team composed of a neonatal nurse practitioner, a neonatologist, a pediatric radiologist, a NICU fellow, residents, a radiology technologist, nurses, and respiratory therapists. The quality components of a neonatal radiograph were derived from a literature review in conjunction with the OSUWMC Radiology Department recommendations. The resultant components included (1) no rotation, (2) flat positioning of infant and head of bed, (3) no artifact (tubes, leads, or other medical equipment within target view), and (4) accurate collimation of the x-ray beam. Accurate collimation was defined as an x-ray beam that was within 1.5 finger widths, circumferentially of the intended target, as recommended by the OSUWMC Radiology Department's practice. A quality radiograph included all 4 components as specified in the electronic medical record (EMR) order. A document with the specific criteria used for evaluating radiographs is included in the Supplemental Materials section. (**See Supplemental Digital Content 1,** which display the radiograph evaluation image criterion 2020, https://links.lww.com/PQ9/A723.)

In this project, we evaluated radiographs for both PICCs and ETTs. The device tip evaluation was primarily outside the scope of this project, as infant position was the primary outcome for these 2 devices. An upper extremity PICC required an infant’s arm to be flexed and held against the ipsilateral chest.^13–15^ A lower extremity PICC required that the infant’s leg be held in a frog-like position, level to the hips.^13,14^ The infant’s neck was to be held in a neutral position when ETT was indwelling.^2,17–19^

We established review criteria to facilitate an objective evaluation of the radiographs for the project group’s providers. We classified the radiographs into 3 groups. The All-X-Ray Group included only chest, abdomen, or baby films. The PICC group consisted of all chest and baby gram radiographs with an indwelling extremity PICC. The ETT group included all chest and baby gram radiographs with an endotracheal tube in place. Because the PICC and ETT groups were subsets of the All-X-Ray Group, there was overlap of radiographs across groups. Each film was reviewed individually and counted as a separate subject of review for each group. Providers within the project group were the radiograph reviewers. During the Baseline Period, the reviewers included 3 neonatal nurse practitioner (NNP) students, 1 NNP, and 3 physicians, one of whom was a pediatric radiologist. During the Intervention and Sustain Periods, the reviewing group consisted of 1 NNP and 2 physicians, who remained consistent across the remaining project periods. The pediatric radiologist did not participate after the Baseline Period due to clinical constraints.

We divided the project into 3 periods: Baseline Period, Intervention Period and Sustain Period. The Baseline Period for the All-X-Ray Group was from October 1 to October 31, 2020, whereas the ETT and PICC subgroups extended from May 1 to October 31, 2020. The Intervention Period started on May 19, 2021, and lasted until March 31, 2022, for all groups. The Sustain Period began June 1 and ended July 31, 2022, for all groups.

**Fig. 1.**
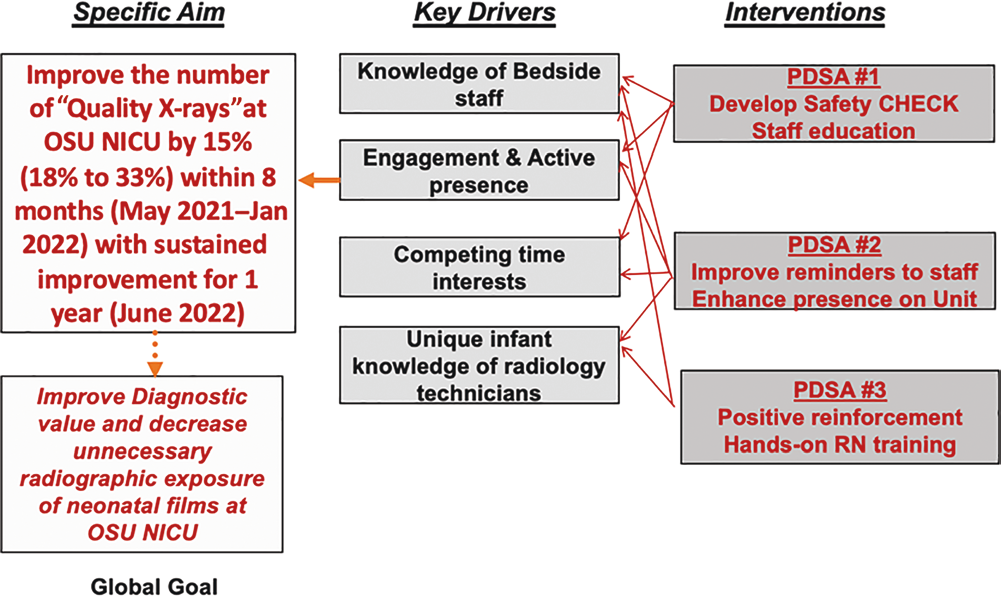
Key driver diagrams outlining project aims and Plan–Do–Study–Act cycles.

Using the Institute for Healthcare Improvement model, a key driver diagram was developed (Fig. 1). The global aim of the project was to increase the frequency of high-quality diagnostic radiographs while minimizing the need for repeat radiographs.

### Intervention 1: Development of the CHECKlist

**Fig. 2.**
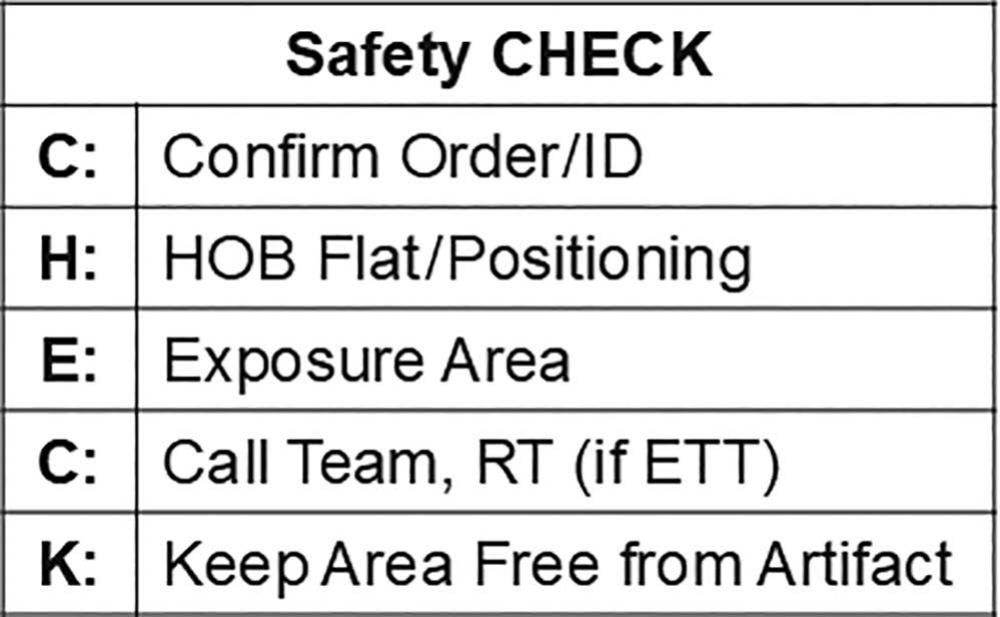
Safety CHECKlist.

The first intervention was the development of a Safety CHECKlist, which included all the components of a quality radiograph (Fig. 2). This CHECKlist outlines how to position an infant for a radiograph by following the components listed in Figure 2. The bedside personnel completed the CHECKlist forms. Pictures of infants with indwelling PICC were posted at the bedsides of infants with indwelling catheters. The reference PICC signs served as reminders for nurses and radiology technologists, as all radiographs were taken using a portable machine at the bedside.

### Intervention 2: Increased Unit Presence

To enhance the awareness and use of the Safety CHECKlist among staff, we strengthened our reminders to nursing through monthly project progression emails. The CHECKlist forms were added to the infant admission packet to increase accessibility. We displayed small CHECKlist signs on EMR computers in the unit, the x-ray machine, and the walls of the NICU pods and hallways. Although not a measured intervention, the providers in the project group (NNP + fellow) offered to be the provider present at the bedside to further increase presence and support.

### Intervention 3: Positive Reinforcement and Education

We provided real-time positive feedback via verbal and written reminders to bedside providers. This feedback was also shared with nurse managers. A radiographic education session was added to all annual registered nurse (RN) skills day sessions in 2022.

### Sustain Period

Safety CHECK signs on the NICU computers and hallways remained posted through April 2022; however, all active interventions and reminders were stopped.

### Statistical Methods

Films with infants in proper position for the indwelling device, PICC or ETT, at baseline, intervention, and sustain are presented as percentages and frequency. We analyzed data in a time series using statistical process control charts using QI Macros. P-charts are used to visualize the proportion of quality diagnostic radiographs per week. Centerlines are recalculated for each observation period, and ±3 sigma variable-width control limits are presented to identify special-cause variation in the frequency of quality diagnostic x-rays.

### Measures

The primary outcome measure was the percentage of quality diagnostic radiographs in each of the 3 groups. Process measures included the percentage of completed CHECKlist forms and the number of weekly staff positive communications. The time to completion of the safety CHECK was documented and analyzed as a balancing measure.

## RESULTS

During the Baseline Period, there were 156 All-X-Ray Group radiographs, 113 PICC group radiographs, and 90 ETT group radiographs with an overlap of 62 films between subgroups. During the Intervention Period, there were 899 All-X-Ray Group radiographs, comprising 243 from the PICC group and 301 from the ETT group, with an overlap of 127 radiographs between the 2 subgroups. During the sustain period, there were 265 radiographs in the All-X-Ray Group, of which 74 were in the PICC group and 116 in the ETT group, with an overlap of 64 radiographs between the subgroups. Inclusive of all groups and overlapping during all periods, a total of 2067 radiographs were included in the analysis, of which 1461 were unique. The CHECKlist intervention denoted provider presence at an infant’s bedside for radiographs 88% of the time.

**Fig. 3.**
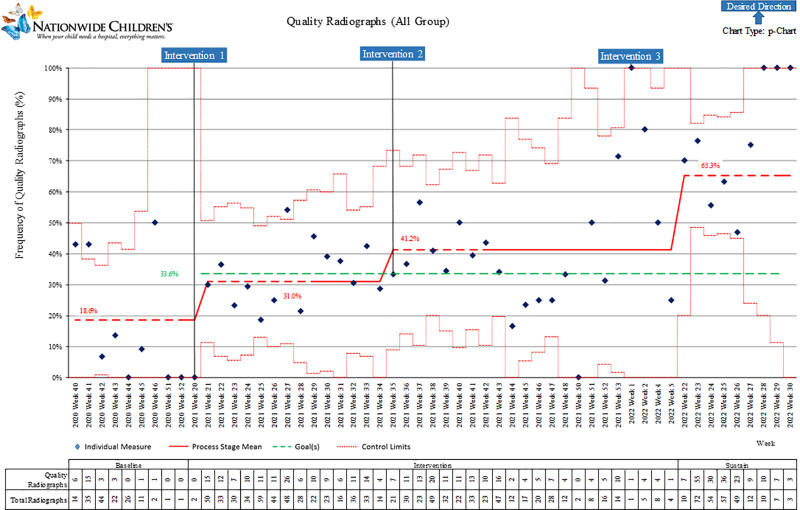
P-chart quality radiographs by week: All-X-Ray Group.

The control chart (P-chart) of the All-X-Ray Group is shown in Figure 3. At baseline, 19% of radiographs were considered quality diagnostic films. Following the CHECKList intervention, we observed an increased improvement to 34%. A second special cause variation can be observed shortly after the second intervention, with an improvement to 41%, followed by a gradual increase over time, resulting in sustained success 65% quality diagnostic film rate at one year.

**Fig. 4.**
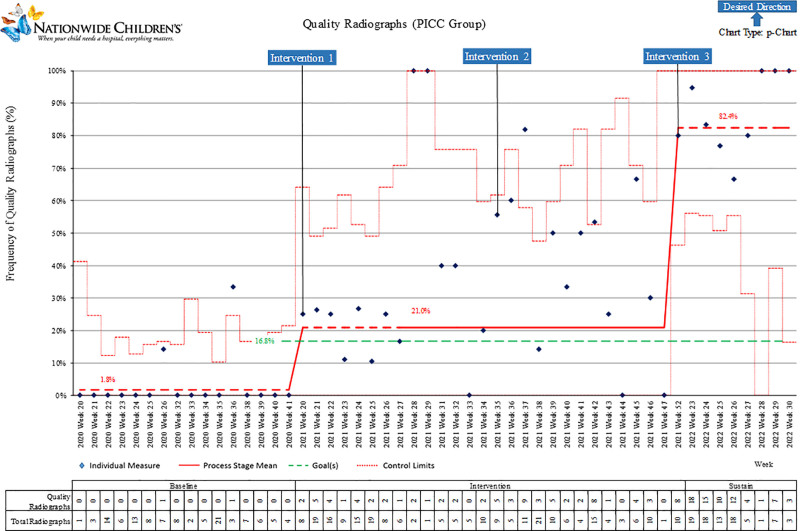
P-chart quality radiographs by week: PICC group.

The control chart (P-chart) of the PICC group is shown in Figure 4. This group had a baseline of fewer than 2% of films that were of quality diagnostic value. Following the CHECKlist implementation, a special-cause variation was observed, with an increase to 34%. The sustain period shows a persistent improvement, resulting in 81% of quality diagnostic radiographs. Our data indicated that infants in the PICC group were not consistently positioned appropriately. As recognition of inconsistent form completion became apparent within the study timeframe, noted by a 30% return rate of paper Safety CHECKlist forms, we integrated verbal reminders for RNs providing appropriate positioning.

**Fig. 5.**
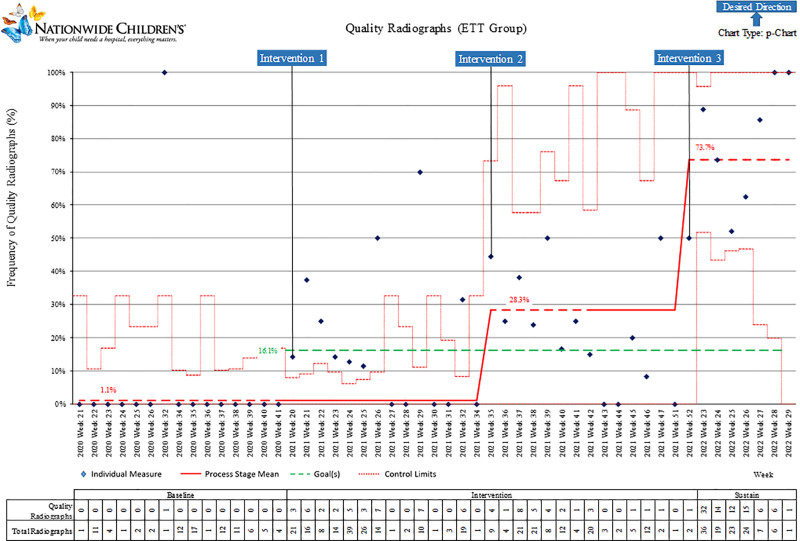
P-chart quality radiographs by week: ETT group.

The ETT group control chart (P-chart) is shown in Figure 5. This group had the lowest baseline of quality diagnostic films at 1%. Improvement is not evident until after the second intervention, with an increase to 24%. An additional increase to 74% occurred during the sustain period.

**Fig. 6.**
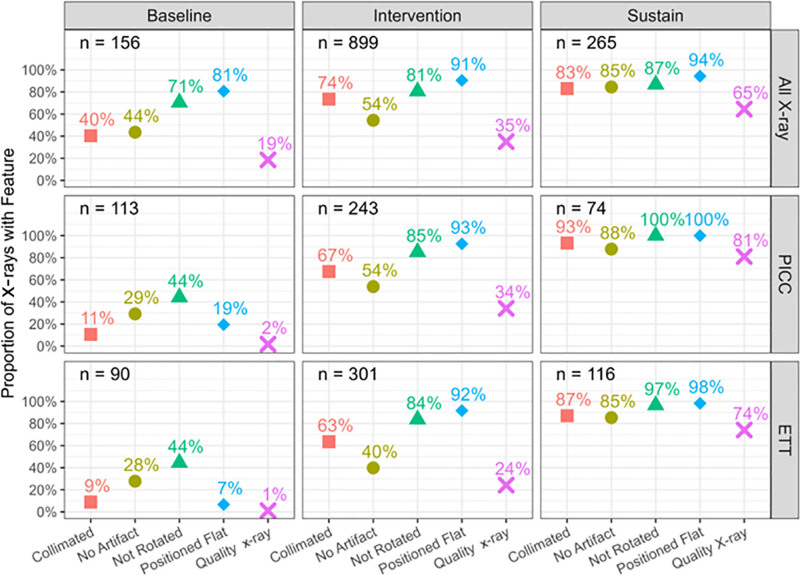
Proportion of radiographs meeting each criterion requirement during the project periods.

Flat positioning demonstrated the highest accuracy across all periods, with collimation and artifact presence denoting the most prominent improvements (Fig. 6). Although decreasing repeat films across the NICU was a target for the project, the statistical analysis did not reveal a significant overall difference in repeat films. For the All-X-Ray Group during the Baseline Period, the median was 1, with the first and third interquartiles at 1 and 3, respectively. Then, in the sustain period for the All-X-Ray Group, the median was 2, and the first and third quartiles were 1 and 3, respectively. The results were similar for the other 2 subgroups. The project’s overall effect on repeat films was negligible.

## DISCUSSION

### Summary

This project implemented a pre-x-ray Safety CHECKlist with the specific aim of improving the frequency of high-quality diagnostic films by 15% during a 9-month period. A total of 2,067 neonatal radiographs were evaluated, with 1,461 being unique. The results demonstrated that the improvements exceeded the target of 15% across all groups, with sustained effects evident 1 year later. The project supported a team approach that fosters outcomes. Notably, the consistent improvement throughout the project periods demonstrates staff buy-in for the project.^20^ Like Edison et al,^9^ the project group thought that empowering the NICU staff with the proper radiographic tools for accurate positioning of an infant was an important factor for success. Although the frequency of quality diagnostic films was unknown, the diagnostic quality criteria categories (rotation, flat positioning, no artifact, and accurate collimation) were reported in terms of percentages to the NICU staff and radiology technologists monthly via email and department staff meetings.

### Strengths

This project included a large number of neonatal radiographs (1,461) and exceeded the targeted improvement after 12 months. Each group demonstrated improvement across the Baseline Period through the sustain period (Figs. 3–5). The providers who reviewed the radiographs were experienced, read neonatal radiographs in daily practice, and used the same criteria.

Before the Intervention Period, due to a perceived need to repeat PICC films for correct positioning, the project leads provided staff with written and verbal reminders to minimize repeat films, particularly in cases of incorrect infant positioning. There was no means of determining the impact of the reminders on the PICC group’s outcomes, as the target of the staff recognition was to decrease the number of repeat radiographs. Because the number of quality diagnostic films across periods from the Baseline Period to the Intervention Period was unknown until analysis was completed, the verbal and written reminders may have influenced the greater improvement seen in the PICC group (1.8% to 34% to 81%); however, no data were collected to support or quantify the impact.

### Interpretation

The application of the 4 diagnostic quality criteria in this project led to a substantial improvement in the frequency of quality diagnostic radiographs in the NICU during a 9-month period, which was sustained for 1 year. The criteria used for this project have been shown to have a significant impact on the diagnostic quality of a neonatal film.^2,4,16,19^ Holding an infant to limit the amount of positional rotation is imperative.^2,9^ This project, similar to Motz et al^2^ in 2020, instituted a radiograph standard including infant positioning, which demonstrated a significant improvement in both PICC and ETT quality diagnostic films and a reduction in repeat radiographs for PICC and ETT positioning by using specific QI criteria.

Infant rotation in a radiograph may impact diagnostic interpretation.^9,11,12^ We educated the NICU and radiology staff on proper holding techniques for infants undergoing radiographic examinations. We were able to decrease the amount of infant rotation on radiographs by 16%, 56%, and 53% in the All-X-Ray Group, PICC group, and ETT group, respectively. Although rotation is common and almost unavoidable in the NICU, limiting rotation can facilitate the accuracy of diagnostic interpretation.^21^

There are numerous projects that have focused on reducing the dose of radiation to neonates; however, the recommended practice varies.^22^ Edison et al^9^ evaluated the criteria for a quality diagnostic film, focusing on optimizing safety hazards associated with neonatal radiographs. This included tracking the collimation of the x-ray beam. Through increased staff awareness and a focus on compliance with QI measures, the group was able to minimize radiograph hazards within their respective unit by 84%.

Our project included a pre-x-ray checklist, which confirmed that the EMR x-ray order was also reviewed by others.^9,12^ Edison et al^9^ also included the EMR order for accuracy before the radiograph. Our Safety CHECKlist included monitoring the presence of the medical provider at the bedside to confirm the accuracy of the order in the EMR. The data showed that a provider was present for 88% of the radiographs. NICU providers attended high-risk deliveries and all emergent clinical situations, which may have prevented bedside presence in 100% of patients.

The aim of this project was to ensure the proper positioning of the infant with an indwelling PICC or ETT. Consistent infant position for device tip evaluation is imperative.^2^ Using a standard position to compare serial radiographs is ideal.^13,14^ Sharp et al^14^ reported that PICC tip migration is the most common complication, indicating the need for serial radiographs during PICC infusion therapy.

Maintaining consistent positioning for evaluating ETT tips is vital for appropriate respiratory management. The position of the ETT changes with head flexion and extension. Im et al.^1^ found a significant association between head position and ETT position, reporting that head movement may affect the ETT depth by 0.7 cm. Our project used a neutral head position for intubated infants when evaluating ETT tip position, as recommended in earlier reports.^2,16–19^

### Limitations

The project spanned a 9-month period, during which all radiographs were reviewed based on a neonate’s admission to the NICU, rather than a specific diagnosis. There was no randomization, and neither the participants, reviewers, nor staff members were blinded to the project. The group that reviewed the films differed across the project time periods.

All infants admitted during the study periods had 100% of their eligible films reviewed. The project took place in 1 NICU with at least 2 providers assisting and evaluating radiographs on a routine basis. This may have positively impacted the frequency of quality diagnostic radiographs, thus affecting project outcomes. These 2 providers were not present for all films, nor were they present daily; therefore, their impact on the data is unknown.

Pedersen et al^4^ reported that rotation can be affected by the position of the infant’s pelvis, upper and lower thorax, and head. We examined the symmetry of the clavicles, the alignment of ribs from front to back, and the position of the infant’s spine in relation to the chest as markers of rotation.^12,23^ We did not evaluate the position of an infant’s pelvis. The standard arm position for our babies was over their head, except for those with an upper extremity PICC. A review of the position of an infant’s pelvis may have impacted the results and should be considered in future NICU radiographic projects.

Another safety factor monitored was the number of repeat films, which was inaccurate during the sustain period. This was because the reviewers were unfamiliar with the symbols in the imaging software. They relied on the number of images loaded into the EMR’s imaging software. If a neonate required repeat images, unbeknownst to the reviewers, the actual number of images was not always transmitted into the EMR. The reviewing group noted that the consistency of the images uploaded was not the same for each technologist. And, because the reviewers did not understand that the symbol indicated the number of images per radiograph session until the sustain period, it was unrealistic to repeat all reviewed images for accuracy. The actual number of films repeated may be much higher than reported in the results. This safety element is important as the degree of radiation harm is associated with the number of radiographs over time, and for the NICU population, the actual amount of harm over time is unknown.^6,8,22^

This project educated NICU staff on how to position infants for high-quality diagnostic radiographs. Before this project, education specific to radiograph practice was more than a decade old. Due to high turnover among bedside RNs, management changes, and inconsistent expectations for nursing and radiology technologists, radiograph education was outdated. Coincidentally, a QI project was conducted concurrently in the radiology department and may have positively impacted the NICU project’s outcomes, as confirming patient identification, positioning, and collimation were components in the radiology department’s project.

## CONCLUSIONS

This project reviewed a robust number of radiographs in a level III NICU, demonstrating significant improvements in radiograph diagnostic quality throughout the course of a year. Key interventions included the development of a CHECKlist, staff education, staff recognition, and provider involvement. Improving radiograph quality is a valuable QI project, as minimizing unnecessary radiation exposure in neonates can have long-term health benefits. This project had a meaningful impact on bedside radiographs during this time period. Sustainability relies on ongoing education and consistent support for NICU staff and radiology technologists due to frequent staff turnover.

## ACKNOWLEDGMENTS

We acknowledge Charles Kimble, Quality Strategist at the Center for Clinical Excellence, Nationwide Children’s Hospital, Columbus, OH, for assistance with study figures. We also acknowledge Amanda Miller, BS, respiratory therapist (R), the OSUWMC Radiology Department, for assistance with radiographic requirements for image review.

## Supplementary Material


