# Supplementary material for: Implementation of a Pre-X-Ray Safety CHECK in Neonates
Source: Pediatr Qual Saf. 2025 Dec 23;10(6):e860. doi: 10.1097/pq9.0000000000000860 (PMC13169110; doi:10.1097/pq9.0000000000000860)
Supplement: Supplementary file 1 [file pqs-10-e860-s001.pdf]

# Radiograph Evaluation Image Criterion 2020

| | |
|---|---|
| **Abdomen** | |
| **Anatomy:** | Is all the required anatomy included (diaphragms through pubic symphysis and left and right margins of abdomen imaged) |
| **Collimation:** | |
| **Vertical:** | 1.5 finger width allowed above diaphragms and below pubic symphysis |
| **Horizontal:** | 1.5 finger width allowed on sides |
| **Artifacts:** | Were all external artifacts that are able to be moved out of the required anatomy? Leads/Tubing/Temperature Probe/Soiled Diaper/ST of arms, etc. |
| **Rotation:** | Is there any rotation seen on the image? Looking at pelvis, spine, ribs etc. |
| | |
| **Chest** | |
| **Anatomy:** | Is all the required anatomy included (top of shoulders to L1 and through margins of widest part of the chest) |
| **Collimation:** | |
| **Vertical:** | Top of shoulders to L1 (1.5 finger width allowed above shoulders and lower limit is L2) *L1 is approximately at the elbow level when arms are straight at the side. |
| **Horizontal:** | Lateral margin of shoulders, widest part of chest (with 1.5 finger width allowed on sides) |
| **Artifacts:** | Were all external artifacts that are able to be moved out of the required anatomy? Leads/Tubing/Temperature Probe/ST of arms, etc. |
| **Rotation:** | Is there any rotation seen on the image? Looking at ribs, spine, clavicles, etc. |
| | |
| **Babygram** | |
| **Anatomy:** | Is all the required anatomy included (top of shoulders to pubic symphysis and through margins of widest part of the chest/abdomen) |
| **Collimation:** | |
| **Vertical:** | 1.5 finger width allowed above shoulders and below pubic symphysis |
| **Horizontal:** | 1.5 finger width allowed on sides |
| **Artifacts:** | Were all external artifacts that are able to be moved out of the required anatomy? Leads/Tubing/Temperature Probe/Soiled Diaper/ST of arms, etc. |
| **Rotation:** | Is there any rotation seen on the image? Looking at pelvis, spine, ribs, clavicles, etc. |
| | |
